# Prevalence of Cardiovascular Functional Anomalies in Large-for-Gestational-Age (LGA) Fetuses by Fetal Echocardiography

**DOI:** 10.3390/jcm14134500

**Published:** 2025-06-25

**Authors:** Łucja Hanna Biały, Oskar Sylwestrzak, Julia Murlewska, Łukasz Sokołowski, Iwona Strzelecka, Maria Respondek-Liberska

**Affiliations:** 1 Students’ Prenatal Cardiology Scientific Group, Medical University of Łódź, 92-213 Łódź, Poland; 2 Department of Prenatal Cardiology, Polish Mother’s Memorial Hospital Research Institute, 93-338 Łódź, Poland; 3 Department of Obstetrics and Gynecology, Polish Mother’s Memorial Hospital Research Institute, 93-338 Łódź, Poland; 4 Department of Fetal Malformations Diagnosis and Prevention, Medical University of Łódź, 90-419 Łódź, Poland

**Keywords:** LGA, neonatal macrosomia, echocardiography, ductal constriction, prenatal cardiology

## Abstract

**Background/Objectives**: The aim of this study was to determine the prevalence of functional cardiovascular anomalies detected on fetal echocardiography in third-trimester large-for-gestational-age (LGA) fetuses, who were subsequently born as macrosomic newborns with a birth weight exceeding 4000 g. **Methods**: A retrospective study was conducted on 1002 fetuses examined during the third trimester at our fetal cardiology center between 2018 and 2024. All fetuses were classified as having “normal heart anatomy” (NHA). Statistical analysis was performed using Microsoft Excel 2024, Statistica 13.1, and EasyMedStat (version 3.37.1). A *p*-value of <0.05 was considered statistically significant. **Results**: The 1002 fetuses were divided into two groups. The study group (NHA-LGA) consisted of 167 fetuses born with a weight of >4000 g and the control group (NHA-AGA) was made up of 835 fetuses with a birth weight between 2500 and 4000 g. In the NHA-LGA group, 24 fetuses (14.4%) experienced ductal constriction (DC), while in the NHA-AGA group, it was 11 (1.3%) fetuses (*p* < 0.00001). Myocardial hypertrophy was observed in 30 fetuses (18.0%) in the NHA-LGA group versus 72 (8.6%) in the NHA-AGA group (*p* < 0.0003). Additionally, cardiomegaly was noted in 95 fetuses (11.4%) in the NHA-LGA group, compared to 37 (4.4%) in the NHA-AGA group (*p* < 0.0004). **Conclusions**: LGA fetuses with normal heart anatomy may present with functional cardiovascular anomalies, including ductal constriction, myocardial hypertrophy, and cardiomegaly. In our cohort, such anomalies were identified in up to 51% of cases. These findings suggest that targeted fetal echocardiographic screening in macrosomic fetuses could be clinically valuable, even in the absence of structural heart defects, and may aid in the early identification of functional cardiac alterations that could impact perinatal management.

## 1. Introduction

Based on estimated fetal weight (EFW) measured during prenatal ultrasound, fetuses can be classified into four groups: small for gestational age (SGA), appropriate for gestational age (AGA), and large for gestational age (LGA), and—based on longitudinal assessment and growth charts—a fourth group currently recognized as fetal growth restriction (FGR). LGA is typically defined as an EFW or abdominal circumference (AC) above the 90th percentile for gestational age [1]. LGA fetuses are often identified as those likely to be macrosomic, meaning those with a birth weight greater than 4000 g [1,2].

Certain maternal conditions increase the risk of fetal macrosomia. For example, maternal obesity and pregestational diabetes (PGDM) are associated with a higher likelihood of giving birth to a macrosomic infant [3,4].

Accurately predicting whether a fetus is LGA is particularly important, as LGA status is linked with a higher risk of complications such as emergency cesarean section, postpartum hemorrhage, maternal anal sphincter injury, neonatal shoulder dystocia, brachial plexus injury, clavicle or humerus fractures, and birth asphyxia [5]. These findings have clinical relevance and potential implications for prenatal care and decision-making.

Despite the availability of numerous ultrasound markers for fetal macrosomia, there is currently a lack of data regarding fetal heart evaluation in these cases—particularly in fetuses with normal heart anatomy during the third trimester.

## 2. Materials and Methods

From all 4471 fetuses examined at our prenatal cardiology center between 2018 and 2024, this retrospective study was based on a selected group of 1002 fetuses who underwent fetal echocardiography in the third trimester of pregnancy.

Inclusion criteria:

- Fetal echocardiography performed between 28 + 0 and 39 + 0 weeks of gestation;
- Fetuses classified as having normal heart anatomy (NHA) during fetal echocardiography at our center;
- Singleton pregnancies;
- Full-term birth, defined as≥ 37 + 6 weeks of gestation;
- The availability of postnatal follow-up.

Exclusion criteria:

- Fetal echocardiography performed before 28 + 0 weeks of gestation;
- Fetuses diagnosed with congenital heart defects (CHDs) during fetal echocardiography at our center;
- Extracardiac malformations (ECMs) diagnosed during fetal ultrasound;
- Fetuses with prenatally or postnatally confirmed genetic abnormalities (e.g., trisomy 21);
- Multiple pregnancies;
- Preterm birth;
- A lack of postnatal follow-up.

All diagnoses were evaluated and confirmed by a senior physician. Two groups were established:

**Study group (NHA-LGA):** Fetuses with normal heart anatomy and macrosomic neonates (birth weight > 4000 g), *n* = 167;

**Control group (NHA-AGA):** Fetuses with normal heart anatomy and a birth weight between 2500 and 4000 g, *n* = 835.

**Figure 1:**
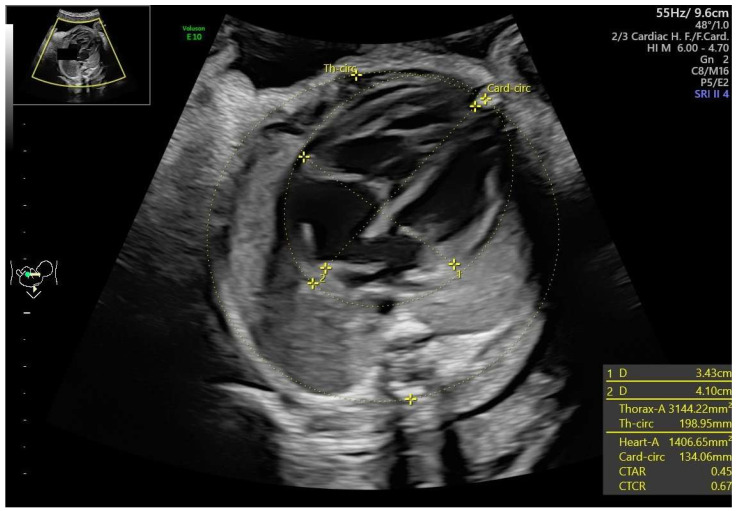
Fetal cardiomegaly, HA/CA (CTAR) 0.45 (29 weeks of gestation).

**Figure 2:**
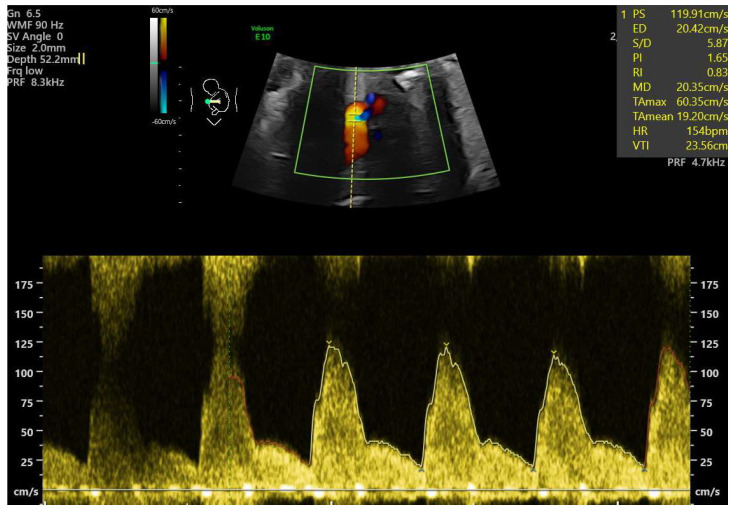
Fetal ductal constriction with a maximal velocity (PSV) of 119.91 cm/s and a pulsatility index of 1.65 in pulsed-wave Doppler. Turbulent flow in the ductus arteriosus (DA) with aliasing in color Doppler signifies turbulence inside the vessel and increased maximal velocity in the DA (33 weeks of gestation).

**Figure 3:**
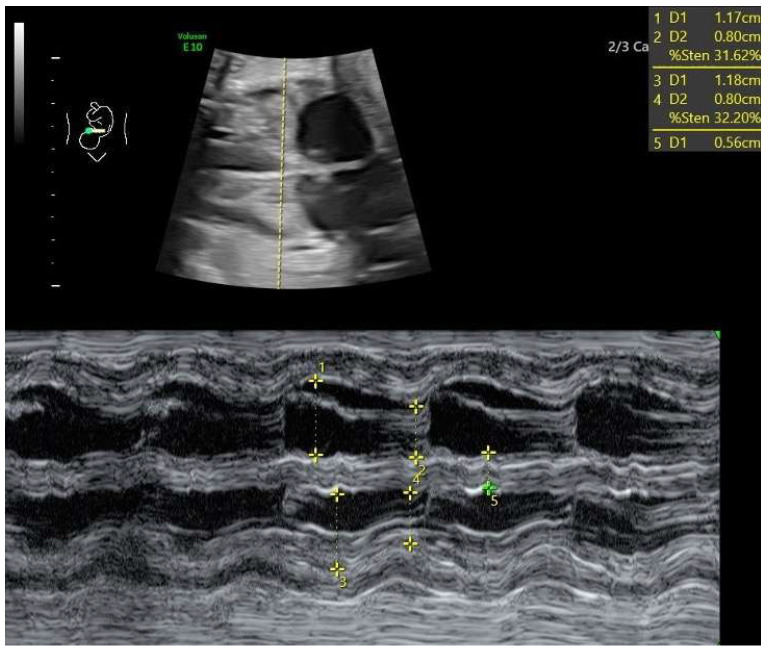
Fetal myocardial hypertrophy, septal thickness 5.6 mm in M−mode, (35 weeks of gestation). The thickness was determined in diastole using the M−mode technique in the maximal enlargement possible for the maximal accuracy with the apex of the heart at the 3rd or 9th “hour”.

Fetal functional cardiovascular abnormalities, maternal conditions, and neonatal outcomes were analyzed. Functional cardiac abnormalities included:

- Cardiomegaly, defined as a heart area-to-chest area ratio > 0.42 [6,7] (Figure 1).
- Ductal constriction (DC), defined as a peak systolic velocity (PSV) > 1.4 m/s or >95th percentile for gestational age, a peak diastolic velocity > 35 cm/s or >95th percentile, or a pulsatility index (PI) < 1.9 [8,9]. The measurement was taken by visualizing the DA at the 12th or 6th “hour” (+/− 30 degrees), which was performed by seeing the ductal arch in the long-axis view. Fetal slow movements or hiccups were accounted for and if they occurred the measurement was taken more times during the examination (Figure 2).
- Myocardial hypertrophy, defined as an interventricular septal thickness > 4.5 mm in diastole, regardless of gestational age, with the apex of the heart at the 3rd or 9th “hour”, so that the M-mode is performed at 90 degrees [10] (Figure 3).
- Tricuspid regurgitation (TR), detected via color or pulsed-wave Doppler, with a peak systolic velocity > 1.5 m/s and a duration > 80 ms [11].
- Pulmonary regurgitation (PR), seen in color or pulsed-wave Doppler at the right ventricular outflow tract (RVOT) or pulmonary trunk [12,13].
- Aortic regurgitation (AR), detected at the left ventricular outflow tract (LVOT) [13].
- Mitral regurgitation (MR), detected at the mitral valve [13].
- Pericardial effusion, defined as fluid > 3 mm [13].
- Disproportion in the four-chamber view (4CV), defined as a ≥2 mm disparity between atria, ventricles, or both [13].
- A bright spot in the left or right ventricle [13].
- Fetal arrhythmia, detected via M-mode or color Doppler [13].
- Tricuspid monophasic flow, defined by the absence of distinguishable E and A waves [13].
- Foramen ovale (FO) abnormalities, such as atypical flow (bilateral or left-to-right in the third trimester) or the presence of a “spinnaker” FO (long, supple FO valve) [13].
- Umbilical cord around the neck, noted due to its potential to mimic arrhythmia or disproportion in 4CV [13].

All fetuses had previously undergone first- and second-trimester echocardiographic evaluations confirming normal heart anatomy. Third-trimester scans were performed at a mean of 33 weeks, following the Polish Recommendation [14], with a focus on detecting functional abnormalities.

Maternal conditions included chronic illnesses such as diabetes mellitus (DM) and obesity at the start of pregnancy. DM was further classified into:

- Pregestational diabetes mellitus (PGDM);
- Gestational diabetes mellitus (GDM):
  ∘ G1: Diet-controlled;
  ∘ G2: Requiring insulin therapy.

Obesity was defined as a BMI > 30, measured prior to pregnancy.

Neonatal outcomes were assessed based on:

- Birth weight;
- Gestational age and mode of delivery;
- Apgar score;
- A need for phototherapy due to hyperbilirubinemia;
- Neonatal sex.
- A macrosomic neonate was defined as one with a birth weight > 4000 g.

The ultrasound machine Voluson E10 with a 3–6 MHz probe was used to assess fetal weight and perform the fetal echocardiography examination. Before the data were introduced to the dataset, they were reviewed and verified by one investigator, a co-author.

For statistical analysis, the Microsoft Excel 2024, Statistica 13.1, and EasyMedStat (version 3.37.1) programs were used. The characteristics of continuous variables were presented as arithmetic means and SD, or medians and lower and upper quartiles. The statistical characteristics of qualitative variables were presented in the form of numerical and percentage distributions. The qualitative variables were compared between groups using Pearson’s chi-square test (*p* < 0.05). For assessing the relationship between myocardial hypertrophy, cardiomegaly, and the explanatory variables—obesity and diabetes mellitus (DM)— a multivariate logistic regression was performed. Data were checked for multicollinearity with the Belsley–Kuh–Welsch technique. The heteroskedasticity and normality of the residuals were assessed, respectively, by the White test and the Shapiro–Wilk test. A *p*-value < 0.05 was considered statistically significant.

## 3. Results

### 3.1. Maternal Abnormalities

Maternal age did not significantly differ between the two groups. For the NHA-LGA group, the median age was 32 (28–35) years; whereas in the NHA-AGA group median maternal age was 31.0 (28–36) years (*p* > 0.05, Mann–Whitney test), (Table 1).

**Figure 4:**
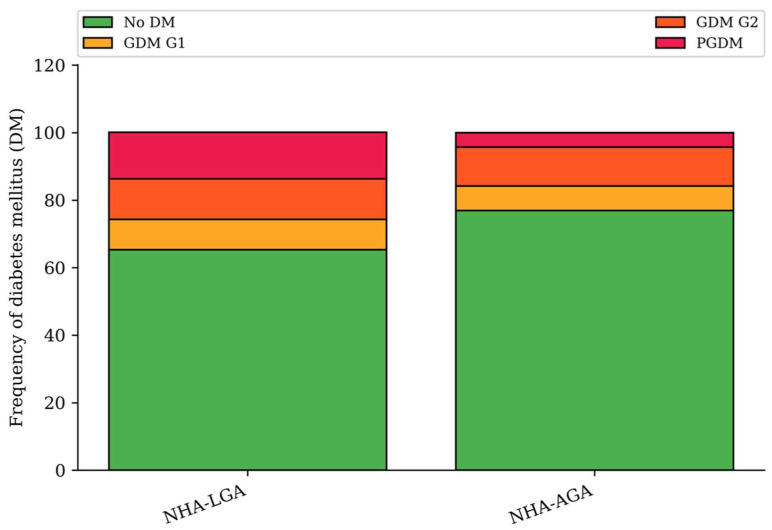
Frequency of diabetes mellitus in the NHA-LGA and NHA-AGA groups (*p* < 0.001).

Diabetes mellitus (DM) affected 58 (34.7%) of the women in the NHA-LGA group and 193 (23.1%) in the NHA-AGA group, (*p* < 0.002). In the NHA-LGA group, 23 (13.8%) of the women suffered from pregestational diabetes mellitus (PGDM) and 35 (21.0%) from gestational diabetes (GDM), which included 15 (9.0%) of the G1 type, where glucose levels were controlled via diet and 20 (12.0%) with the G2 type, which requires insulin treatment. In the NHA-AGA group this was as follows: 36 (4.3%) had PGDM, 61 (7.3%) suffered from G1 type GDM, and 96 (11.5%) had G2 type GDM (*p* < 0.001) (Figure 4). Obesity occurred in 30 (18.0%) of the pregnant women in the NHA-LGA group; whereas, in comparison, in the NHA-AGA group it was 71 (8.5%) (*p* < 0.001).

### 3.2. Functional Abnormalities

Statistical differences (chi-square test, *p* < 0.05) were related to ductal constriction (*p* < 0.001), myocardial hypertrophy (*p* < 0.001), and fetal cardiomegaly (*p* = 0.001). Using Pearson’s chi-square test (*p* < 0.05) there were no statistical differences between the NHA-LGA and the NHA-AGA groups for tricuspid regurgitation (TR), pericardial effusion (PE), disproportion in 4CV, the presence of a bright spot, the prevalence of fetal arrhythmia, tricuspid monophasic flow, foramen ovale abnormal flow (defined as bilateral of left–right flow in the 3rd trimester), the prevalence of pulmonary insufficiency, the presence of foramen ovale as spinnaker or mitral/aortic insufficiency, and nuchal cord (Table 2).

As diabetes is usually connected to fetal myocardial hypertrophy and cardiomegaly, in multivariate analysis, obesity (OR = 1.9, [0.91; 3.96], *p* = 0.0859) and DM (OR = 1.32, [0.73; 2.39], *p* = 0.3598) were not associated with the rate of fetal cardiomegaly. However, in the case of myocardial hypertrophy, DM (OR = 2.41, [1.56; 3.7], *p* < 0.0001) was associated with higher rates of hypertrophy, although obesity (OR = 1.07, [0.56; 2.03], *p* = 0.8388) was not associated with its rate.

**Figure 5:**
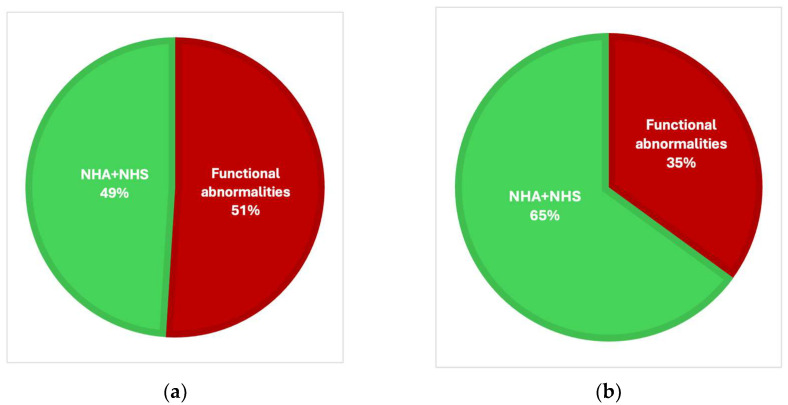
Functional abnormalities in the NHA-LGA (**a**) and the NHA-AGA (**b**) groups, (*p* < 0.001). Functional abnormality was defined as at least one of the following: DC, hypertrophy, cardiomegaly, TR, pericardial effusion, disproportion, bright spot, arrhythmia, tricuspid monophasic flow, FO abnormal flow, pulmonary valve insufficiency, FO spinnaker, MV insufficiency, aortic valve insufficiency, polyhydramnios, ascites, and the umbilical cord wrapped around the fetus’ neck.

Overall, in the NHA-LGA group, only 82 (49.1%) of the fetuses were free from any functional abnormalities; whereas, in the NHA-AGA group, the percentage of fetuses free from any abnormalities was 540 (64.7%) (*p* < 0.001) (Figure 5).

### 3.3. Neonatal Outcome

The neonates were delivered at a median of 39.3 (38.4–40.3) weeks of gestation in the NHA-LGA group and 39.2 (38.3–40.0) in the NHA-AGA group; *p* > 0.05. 119 (71.3%) of neonates in the NHA-LGA group were delivered via cesarean section, in comparison to the NHA-AGA, where it was 451 (54.0%) (*p* < 0.001). The proportion of male and female groups were, respectively, 104 (62.3%) and 63 (37.7%) in the NHA-LGA group, and 420 (50.3%) and 415 (49.7%) in the NHA-LGA group (OR = 0.61; CI [0.44; 0.86]; *p* = 0.006). In the NHA-LGA group, the neonates received a median Apgar score of 10.0 (9–10), compared to 10.0 (9–10) in the NHA-AGA group (*p* = 0.045).

An increased bilirubin level, requiring phototherapy in the NHA-LGA group, occurred in 54 (32.3%) of cases; whereas, in the NHA-AGA group it was 199 (23.8%), (OR = 1.53; CI [1.06; 2.19]; *p* = 0.027). The median levels of bilirubin in the NHA-LGA were 13.8 (12.3–15.6) and in the NHA-AGA group they were 13.35 (12.4–14.5), *p* > 0.05.

The neonates in the NHA-LGA group were hospitalized for a median of 5.0 (3.0–6.5) days after birth, compared to the NHA-AGA group where the median was 4.0 (3.0–5.0), *p* < 0.001.

## 4. Discussion

Assessing fetal growth is one of the primary measurements performed during an obstetric ultrasound examination. The standard estimated fetal weight (EFW) calculation includes the biparietal diameter (BPD), head circumference (HC), abdominal circumference (AC), and femur length (FL) [1]. Currently, the prediction rate for large-for-gestational-age (LGA) fetuses—those with an EFW above the 90th percentile—is approximately 65% at 35 + 0 to 36 + 6 weeks of gestation [15]. Therefore, there is still room for improvement in predicting neonatal macrosomia.

In borderline cases, additional measurements may be helpful. One such measurement is the interventricular septal thickness (IVST). Szmyd et al. [10] reported that IVST, when measured in M-mode during diastole, serves as an additional predictor of fetal macrosomia, with a cut-off value of 4.7 mm.

Beyond fetal biometry, fetal well-being can also be evaluated through fetal echocardiography. In our center, routine third-trimester examinations focus not only on cardiac anatomy but also on heart function [14]. To assess fetal cardiovascular efficiency, the Cardiovascular Profile Score (CVPS) can be used. This score incorporates venous Doppler assessments (including the ductus venosus and umbilical vein), heart size, cardiac function (noting the presence of tricuspid and/or mitral regurgitation), and arterial Doppler in the umbilical artery. A CVPS below 7 points is associated with increased fetal mortality [16].

Numerous cardiovascular functional abnormalities can be observed in the third trimester, including premature constriction of the ductus arteriosus and foramen ovale. In our retrospective study, we demonstrated that macrosomic neonates exhibited a higher incidence of functional abnormalities detected during fetal echocardiography, particularly ductal constriction (DC), myocardial hypertrophy, and cardiomegaly.

Ductal constriction is of particular clinical importance as it may be associated with a range of additional functional abnormalities such as cardiomegaly; right ventricular overload; dilation of the right atrium (RA), right ventricle (RV), and pulmonary artery; right ventricular hypertrophy and dysfunction; and tricuspid and pulmonary valve regurgitation [8,9]. For this reason, assessment of the fetal ductus arteriosus is a crucial component of fetal echocardiography in the third trimester, especially in cases of suspected fetal macrosomia. According to our findings, the incidence of DC was significantly higher in the NHA-LGA group than the NHA-AGA group (*p* < 0.00001).

Given these findings, pregnant women carrying a potentially macrosomic fetus should be advised to follow a recommended diet that excludes specific foods and beverages associated with an increased risk of ductal constriction. These include green tea [17]; black, herbal, and mate teas; wine; dark chocolate; certain fruits (such as oranges, grapes, berries, and prunes); natural fruit juices; olive and soy oils; purple onion; green herbs; tomatoes [18]; and other products rich in polyphenols [19,20,21].

In addition to dietary considerations, patients should be informed about the potential side effects of certain medications. For example, non-steroidal anti-inflammatory drugs (NSAIDs) [22,23,24,25], by inhibiting COX-1 and COX-2 receptors, suppress the biosynthesis of prostaglandins, prostacyclins, and thromboxanes—including prostaglandin E2, which is essential for maintaining ductal patency during pregnancy. Other medications known to cause ductal constriction include indomethacin [26,27,28] and eflornithine [29].

At this point, we can wonder why it is that LGA fetuses experience DC more frequently. One theory is that accelerated growth in these fetuses during the final weeks of gestation may lead to the elongation and kinking of the ductus arteriosus, resulting in segmental narrowing. This process may mimic the physiological postnatal closure of the ductus arteriosus, which typically occurs within the first 12 to 24 h after birth [30,31].

The prenatal diagnosis of DC is a significant finding during fetal echocardiography as it can be associated with complications during the neonatal period. After birth, DC has been linked to an increased risk of respiratory distress. Alvarez et al. [8] reported that 18–28% of such cases can lead to persistent pulmonary hypertension in the newborn (PPHN). Furthermore, it has been observed that neonates with a prenatal diagnosis of DC in the third trimester are more likely to experience neonatal hyperbilirubinemia [32].

Our study also confirmed a higher incidence of septal hypertrophy and fetal cardiomegaly, both of which are known to be associated with maternal diabetes mellitus (DM) [33,34,35]. However, despite these observable cardiac functional abnormalities in utero, a meta-analysis by Depla et al. [34] concluded that the long-term postnatal consequences of these findings remain unclear.

The significantly higher prevalence of both PGDM and GDM in the NHA-LGA group compared to the NHA-AGA group aligns with existing evidence linking maternal hyperglycemia to increased fetal growth and macrosomia [36,37]. Maternal diabetes results in elevated glucose levels crossing the placenta, which stimulates fetal insulin production, a potent growth factor [38], leading to increased fat deposition and accelerated somatic growth in the fetus. This mechanism explains why fetuses of diabetic mothers are more likely to be LGA and subsequently macrosomic. Moreover, the distinction between GDM types (G1 and G2) reflects varying degrees of glucose control and insulin dependence, both of which may impact fetal growth differently. These findings highlight the importance of strict maternal glycemic control to mitigate risks associated with fetal macrosomia and related cardiovascular changes.

Another analyzed abnormality that can be seen both in association with DC and as an isolated incident, was tricuspid regurgitation (TR). In our study, TR was present in 15% of fetuses in the NHA-LGA group (*p* > 0.05). Although it usually occurs in about 6.8% of singleton fetuses as mostly an individual finding [11]. The postnatal consequences of TR were studied by Respondek-Liberska et al. [39] and it was proven that in the case of a neonate with a prenatal history of TR it can influence the outcome of a born-at-term neonate, as it was related to mild neonatal hyperbilirubinemia. In the mentioned study, the authors suggested that it may be because of the primarily impaired liver function, due to intrauterine mild infection, that the TR occurs during fetal life. After birth, in the 4th or 5th day of life, neonates presented with a mean bilirubinemia of 11 mg/dL. In the group without TR, only 8% of neonates presented with higher levels of bilirubin; whereas, in the group with TR it was 46% (*p* < 0.0005).

Fetal functional abnormalities are common findings of the 3rd-trimester echocardiography examination. In our study group only 35% of fetuses had not experienced any functional abnormalities, and in the control group it was 53% (*p* < 0.002). As we are a referral center for fetal cardiology and we provide a second opinion for any question marks from obstetricians after their routine ultrasound study, this may explain why in the control group the incidence of functional abnormalities was as high as 47%. The main criteria for the control group were only birth weight and normal heart anatomy.

We can only speculate as to why LGA fetuses frequently present with cardiac dysfunction. It may be due to the increased metabolic demands and altered hemodynamic conditions associated with excessive growth. In particular, fetal macrosomia—often linked to maternal diabetes or obesity—results in chronic exposure to elevated glucose and insulin levels, which can promote myocardial hypertrophy and altered cardiac remodeling. Later on, they can lead to an impairment of ventricular compliance and diastolic function. Additionally, the increased blood volume and cardiac output to meet the metabolic needs of an LGA fetus may lead to volume overload, contributing to functional changes such as ductal constriction or valve regurgitation. These structural and functional alterations can reduce cardiac efficiency, potentially increasing the risk of perinatal complications. Another hypothesis is that fetal functional cardiac abnormalities may be related not only to fetal excessive growth, but also to an increased placental volume/thickness. This subject is a topic of a separate study by our group.

This study has several strengths, including a large sample size and standardized fetal echocardiography performed by experienced clinicians in a fetal cardiology center. The focus on fetuses with normal heart anatomy and thorough postnatal follow-up adds to the reliability of the findings. However, limitations include its retrospective single-center design, which may affect generalizability. Although echocardiographic evaluations were consistently interpreted by a single experienced physician—reducing inter-observer variability—this also introduces the potential for interpretation bias. Functional anomalies were assessed only in the third trimester, possibly missing earlier changes. Additionally, some maternal factors were not fully controlled, and the lack of long-term follow-up limits understanding of the clinical significance of the findings.

To date, there have been no analyses concerning functional abnormalities in LGA fetuses. To our knowledge this is the very first observation of a connection between ductal constriction and LGA. Therefore, if there is a suspicion of LGA, the velocity and PI of the ductus arteriosus should be measured.

## 5. Conclusions

LGA fetuses with normal heart anatomy may present with functional cardiovascular anomalies, including ductal constriction, myocardial hypertrophy, and cardiomegaly. In our cohort, such anomalies were identified in up to 51% of cases. These findings suggest that targeted fetal echocardiographic screening in macrosomic fetuses could be clinically valuable, even in the absence of structural heart defects, and may aid in the early identification of functional cardiac alterations that could impact perinatal management.

## Figures and Tables

**Table 1:** Maternal factors in the NHA-LGA and NHA-AGA groups.

Maternal Information	NHA-LGA (*n* = 167)	NHA-AGA (*n* = 835)	*p* Value
Maternal age (median, Q1–Q3)	32.0 (28–35)	31.0 (28–36)	>0.05
Gestational age at exam (median, Q1–Q3)	33.0 (30.0–35.2)	33.5 (30.6–36.0)	0.01
Pregestational diabetes mellitus (*n*, %)	23 (13.8%)	36 (4.3%)	<0.001
Gestational diabetes mellitus (*n*, %)	35 (21.0%)	159 (18.8%)	>0.05
BMI > 30 (*n*, %)	30 (18.0%)	71 (8.5%)	<0.001

**Table 2:** Functional cardiovascular abnormalities in the NHA-LGA and the NHA-AGA groups.

Functional Abnormality (*n*, %)	NHA-LGA (*n* = 167)	NHA-AGA (*n* = 835)	*p* Value
Ductal constriction (DC)	24 (14.4%)	11 (1.3%)	<0.001
Hypertrophy	30 (18.0%)	72 (8.6%)	<0.001
Cardiomegaly	19 (11.4%)	37 (4.4%)	0.001
TR	25 (15.0%)	121 (14.5%)	>0.05
Pericardial effusion	11 (6.6%)	51 (6.1%)	>0.05
Disproportion	11 (6.6%)	51 (6.1%)	>0.05
Bright spot	5 (3.0%)	40 (4.8%)	>0.05
Arrhythmia	3 (1.8%)	13 (1.6%)	>0.05
Tricuspid monophasic flow	4 (2.4%)	10 (1.2%)	>0.05
FO flow abnormal	4 (2.4%)	12 (1.4%)	>0.05
Pulmonary valve insufficiency	2 (1.2%)	4 (0.5%)	>0.05
FO spinnaker	1 (0.6%)	10 (1.2%)	>0.05
MV insufficiency	1 (0.6%)	1 (0.1%)	>0.05
Aortic valve insufficiency	1 (0.6%)	1 (0.1%)	>0.05

## Data Availability

Data are available upon reasonable request.

## Abbreviations

The following abbreviations are used in this manuscript:

LGA	Large for gestational age
NHA	Normal heart anatomy
DC	Ductal constriction
EFW	Estimated fetal weight
SGA	Small for gestational age
AGA	Appropriate for gestational age
FGR	Fetal growth restriction
AC	Abdominal circumference
PGDM	Pregestational diabetes
CHD	Congenital heart defect
ECM	Extracardiac malformation
TR	Tricuspid regurgitation
PR	Pulmonary regurgitation
RVOT	Right ventricular outflow tract
AR	Aortic regurgitation
LVOT	Left ventricular outlet tract
MR	Mitral regurgitation
4CV	Four-chamber view
FO	Foramen ovale
DM	Diabetes mellitus
GDM	Gestational diabetes
PE	Pericardial effusion
IVST	interventricular septal thickness
CVPS	Cardiovascular Profile Score
ECHO	Echocardiography
